# FZD7 is a novel prognostic marker and promotes tumor metastasis via WNT and EMT signaling pathways in esophageal squamous cell carcinoma

**DOI:** 10.18632/oncotarget.19586

**Published:** 2017-07-26

**Authors:** Ting-Ting Cao, Di Xiang, Bei-Lei Liu, Tu-Xiong Huang, Bin-Bin Tan, Chui-Mian Zeng, Zhong-Yuan Wang, Xiao-Yan Ming, Li-Yi Zhang, Guangyi Jin, Feng Li, Jian-Lin Wu, Xin-Yuan Guan, Desheng Lu, Li Fu

**Affiliations:** ^1^ Department of Pharmacology and Cancer Research Centre, School of Medicine, Shenzhen University, Shenzhen, China; ^2^ Department of Clinical Oncology, The University of Hong Kong Faculty of Medicine, Hong Kong, China; ^3^ Wuhan University Shenzhen Research Institute, Shenzhen, China; ^4^ Department of Medical Genetics, School of Basic Medical Sciences, Wuhan University, Wuhan, China; ^5^ State Key Laboratory for Quality Research in Chinese Medicines, Macau University of Science and Technology, Macau, China

**Keywords:** FZD7, ESCC, metastasis, WNT, EMT

## Abstract

Frizzled (FZD) proteins are receptors for secreted WNT proteins and play a critical role in the malignant progression of various cancers. However, the role of human FZD family members in esophageal squamous cell carcinoma (ESCC) was rarely investigated. In this study, we found that the *FZD7* gene was the most commonly up-regulated *FZD* member in ESCC cell lines compared with other *FZDs*. TMA studies further validated that FZD7 protein was up-regulated in 165 of 252 (65.5%) informative ESCC patients and significantly correlated with poor overall survival (P=0.001). Additionally, multivariate Cox regression analysis showed that FZD7 overexpression was an independent prognostic factor for ESCC patients. Ectopic expression of *FZD7* could promote ESCC cell metastasis both *in vitro* and *in vivo*. Under WNT3A stimulation, FZD7 was able to induce the nuclear translocation of β-catenin and activate the downstream targets of WNT/β-catenin signaling, as well as promote epithelial-mesenchymal transition (EMT) potential in ESCC cells. Our study demonstrated for the first time that FZD7 contributes to the malignant progression of ESCC and represents a novel prognostic marker and a potential therapeutic target for ESCC patients.

## INTRODUCTION

With the change of human diet structure and the increasingly serious environment pollution, the high incidence of esophageal cancer (EC) has become an innegligible fact. EC is ranked as the sixth leading cause of death from cancer in the world [1]. The incidence of EC varies internationally. Eastern Asia, Eastern Africa and Southern Africa are areas with highest incidence of esophageal cancer, while Western Africa is the area with lowest incidence [1, 2]. Esophageal squamous cell carcinoma (ESCC) is the predominant subtype of esophageal cancer in China. ESCC patients are frequently diagnosed with lymph node metastasis, which is closely correlated with the poor prognosis of the patients [3, 4]. Current ESCC treatment approaches, including surgery, radiotherapy and chemotherapy, could only get limited efficiency. As a result, palliative therapy is the typical choice of most ESCC patients. Furthermore, acquired resistance against radiotherapy and chemotherapy is a difficult problem to be solved, which frequently results in local tumor recurrence or treatment failure [5]. The only method to improve prognosis and survival rate is to achieve diagnosis and efficient therapy at early stage.

WNT/β-catenin signaling pathway is one of the key pathways involved in tumor metastasis. It is well known that WNTs are secreted glycoproteins involved in carcinogenesis, epithelial-mesenchymal transition (EMT), cell migration, and experimental metastasis of cancer cells [6, 7]. Once binding with the cell surface co-receptors, including frizzled family, the WNT proteins could activate the canonical and non-canonical WNT signaling pathways and lead to the expression of critical transcription factors [8].

As the receptors of WNT proteins, human frizzled (FZD) family consisting of 10 members, have been well studied. Each of the FZD family members contains an N-terminal signal peptide, an extracellular cysteine-rich domain (CRD), a seven-pass transmembrane domain, and an intracellular C-terminal PDZ domain. The CRD enables FZD proteins to interact with WNT proteins, while the PDZ domain interacts with disheveled (Dvl) to transduce downstream WNT signals [9].

To date, a number of studies have demonstrated the roles of FZD family members in human cancers. It has been reported that FZD1 could result in drug-resistance in breast cancer cells and ovarian cancer cells [10, 11]. Overexpression of FZD4 was detected in prostate cancer clinical samples and could induce EMT process [12]. FZD8 was reported to promote migration and invasion in breast cancer cells [13].

Although previous studies reported that FZDs play critical roles in the development and progression of human cancers, the role of human FZDs in esophageal cancer has not been addressed yet. In the present study, *FZD7* was revealed to be the most frequently up-regulated *FZD* member in ESCC cell lines. The protein expression pattern of FZD7 in primary ESCCs was investigated. Metastasis-promoting function of FZD7 was demonstrated by both *in vitro* and *in vivo* assays. The tumor-promoting mechanism of FZD7 was also addressed.

## RESULTS

### *FZD7* is the most frequently up-regulated *FZD* member in ESCC cell lines

**Figure 1:**
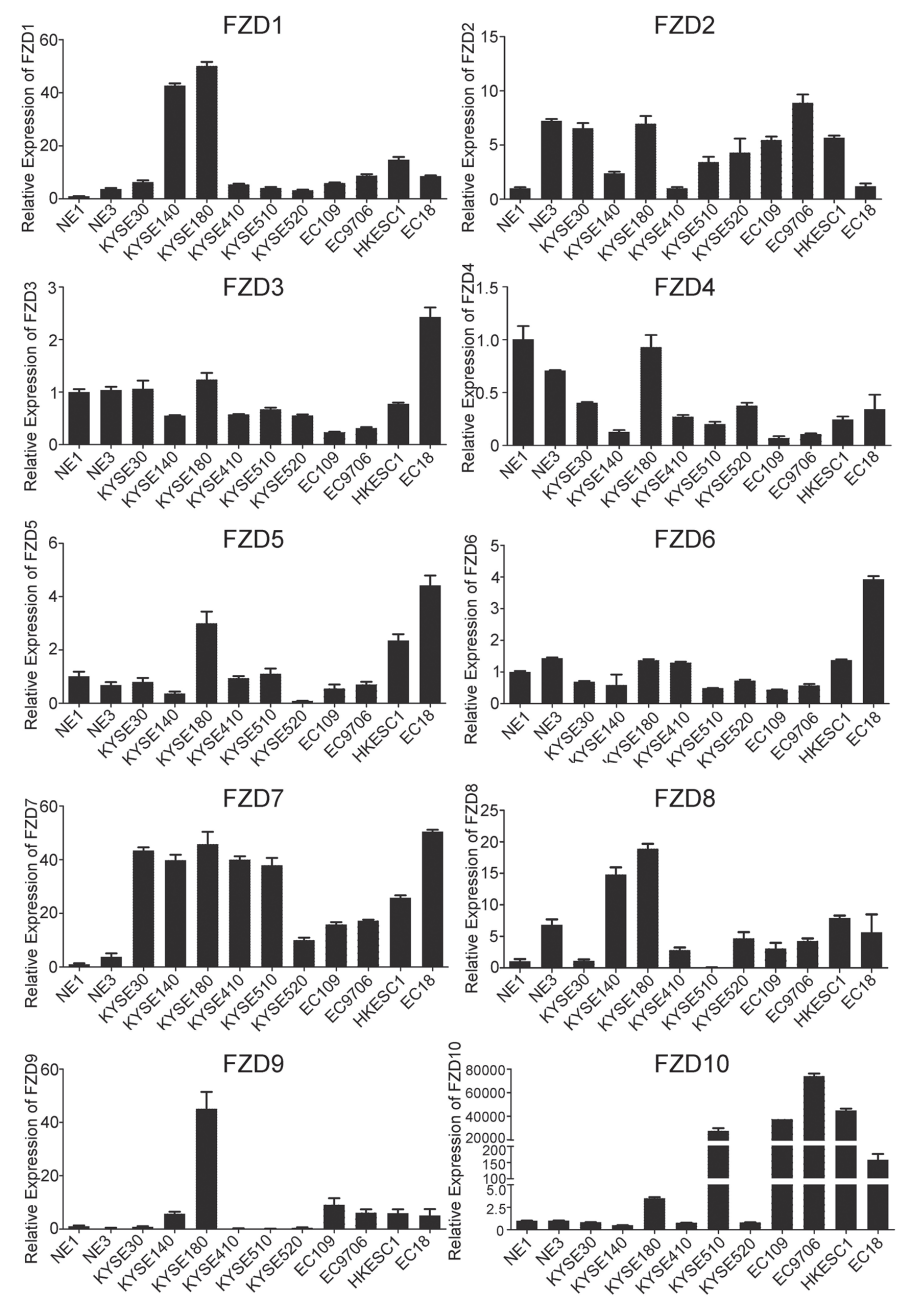
Screening of 10 human *FZDs* mRNA expression in ESCC Quantitative real-time PCR (qPCR) was used to detect *FZD*s transcripts in 10 ESCC cell lines and 2 immortalized normal esophageal epithelial cells. *GAPDH* was used as endogenous control. Data represent the mean±SD derived from three independent experiments.

The frizzled protein family has been well studied in various types of human solid tumors, but rarely reported in ESCC. In this study, we initially screened the transcription level of 10 frizzled family members in 2 immortalized normal esophageal epithelial cell lines and 10 ESCC cell lines. We found that all *FZD* genes were detectable in these cell lines, but the expression levels vary between genes. Only *FZD7* showed frequent up-regulation in all of the 10 ESCC cell lines compared with the two normal esophageal epithelial cells, indicating that *FZD7* may play an important role in ESCC tumorigenesis (Figure 1). Additionally, we detected the protein expression level of FZD7 in these cell lines. The result provided further evidence that FZD7 protein was obviously overexpressed in most of the ESCC cell lines, but was barely detected in NE1, NE3, EC109 and KYSE520 (Supplementary Figure 1). In contrast, the transcriptional levels of *FZD2*, *FZD8* and *FZD10* were only upregulated in several ESCC cell lines, while the expression levels of *FZD3*, *FZD5*, *FZD6* and *FZD9* in most of the ESCC cell lines were similar to the two normal esophageal epithelial cells. Interestingly, *FZD4* was down-regulated in 9 out of the 10 ESCC cell lines, suggesting that this FZD member may have suppressive functions or regulation mechanisms in ESCC.

### Overexpression of FZD7 in primary ESCCs is associated with poor prognosis

**Figure 2:**
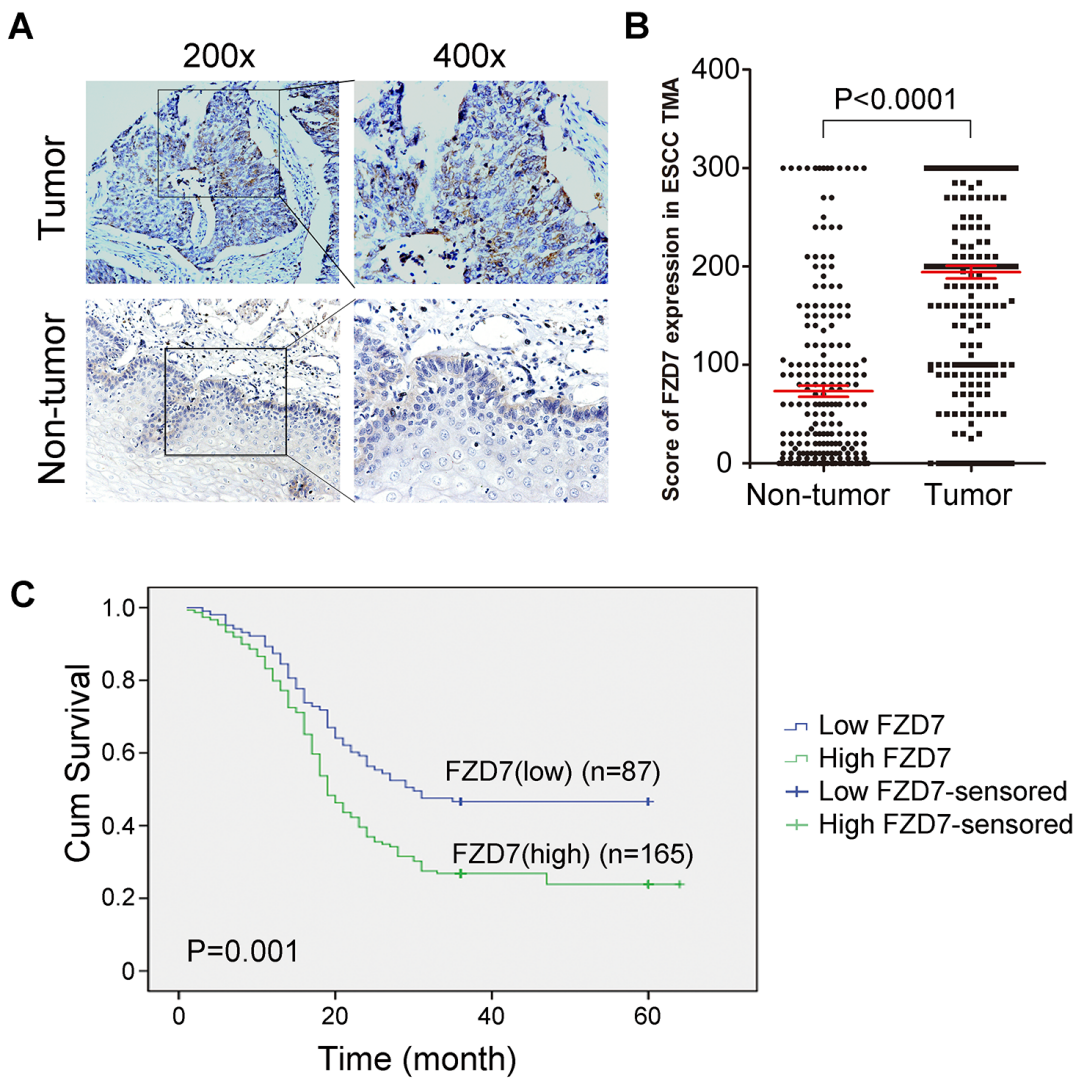
Upregulation of FZD7 in ESCC clinical samples and its prognostic significance in ESCC patients **(A)** FZD7 expression level was detected in ESCC tissues and paired adjacent non-tumor tissues in TMA by immunohistochemistry. **(B)** Scores of FZD7 expression level in non-tumor (NT) versus ESCC tumor for all 252 informative cases on the TMA. **(C)** Kaplan-Meier Survival analysis according to FZD7 expression in 252 informative ESCC patients (log-rank test; *P* = 0.001)

Subsequently, Tissue microarray (TMA)-IHC was performed to examine the protein expression levels of FZD7 in 252 primary ESCCs and their paired adjacent non-tumor tissues. The IHC staining status was evaluated according to the clinical pathological standard. Strong or moderate membrane staining of FZD7 was detected in 165/252 (65.5%) ESCC tissues, whereas no or weak membrane staining of FZD7 was observed in 87/252 (34.5%) of them (Figure 2A). The plot figure showed a high significance in the mean expression level of FZD7 between ESCC tumors and non-tumor samples. (*P*<0.0001; Figure 2B). In addition, Kaplan-Meier analysis showed that FZD7 overexpression was significantly associated with shorter survival time of patients with ESCC. Among the 252 ESCC patients, in 165 cases of strong or moderate FZD7 expression, the median survival time was 19 months and the 5-year survival rate was 6.0%, whereas in 87 cases of low FZD7 expression, the median survival time was 30 months and the 5-year survival rate was 11.2% (*P*=0.001; Figure 2C). To further evaluate whether FZD7 is a prognostic marker for early or late ESCC, we studied the association of FZD7 overexpression with the survival rate in ESCC patients stratified by tumor staging. As shown in the Kaplan-Meier survival curves, PATIENTS WITH high FZD7 expression in early stage had significantly shorter survival TIME (median survival time: 24 months) than those with low FZD7 expression (median survival time: >60 months, *P*=0.0004, Supplementary Figure 2A). However, no difference in survival was found between ESCC patients in advanced stage with (median survival time: 18 months) and without (median survival time: 16 months) FZD7 overexpression (*P*=0.3020, Supplementary Figure 2B), indicating that FZD7 might be a potential prognostic marker for early stage of ESCC patients.

The correlation of FZD7 expression with various clinico-pathologic features was investigated and the result showed that overexpression of FZD7 protein had no relationship with any of the clinical characteristics (Supplementary Table 1). We also performed both univariate and multivariate analyses using COX regression model. By univariable analysis, overexpression of FZD7 (*P*=0.001), poor differentiation (*P*=0.001), presence of lymph node (LN) metastasis (*P*=0.001) and advanced TNM stage (*P*<0.0001) were significant negative prognostic factors for overall survival in ESCC patients (Table 1). Nevertheless, multivariate analysis showed that overexpression of FZD7 (*P*=0.001), poor differentiation (*P*=0.011), advanced TNM stage (*P*<0.0001) were independent prognostic predictors for ESCC patients enrolled in this study (Table 1).

**Table 1:** Association of various factors with overall survival in 252 ESCC patients determined by COX regression model

Variable	Univariate analysis		Multivariate analysis	
	HR ^a^ (95%CI) ^b^	*P* ^c^	HR (95%CI)	*P*
Age (<60 yr vs. ≥60 yr)	1.240 (0.941-1.633)	0.126	1.002 (0.985-1.019)	0.815
Gender (Male vs. Female)	1.130 (0.855-1.495)	0.390	1.066 (0.781-1.456)	0.687
Differentiation (Well/ Medium vs. Poor)	1.633 (1.210-2.205)	**0.001**	1.553 (1.108-2.176)	**0.011**
Lymph node metastasis (absent vs. present)	2.043 (1.547-2.698)	**0.001**	1.095 (0.607-1.977)	0.763
TNM stage (Early vs. Advanced)	2.365 (1.788-3.129)	**<0.0001**	2.258 (1.654-3.084)	**<0.0001**
FZD7 expression (Normal vs. Overexpressed)	1.708 (1.234-2.363)	**0.001**	1.709 (1.235-2.365)	**0.001**

a HR: hazard ratio for death.

b CI: confidence interval.

c P < 0.05 was considered statistically significant (in bold).

### Overexpression of FZD7 could promote esophageal cell mobility and metastasis

**Figure 3:**
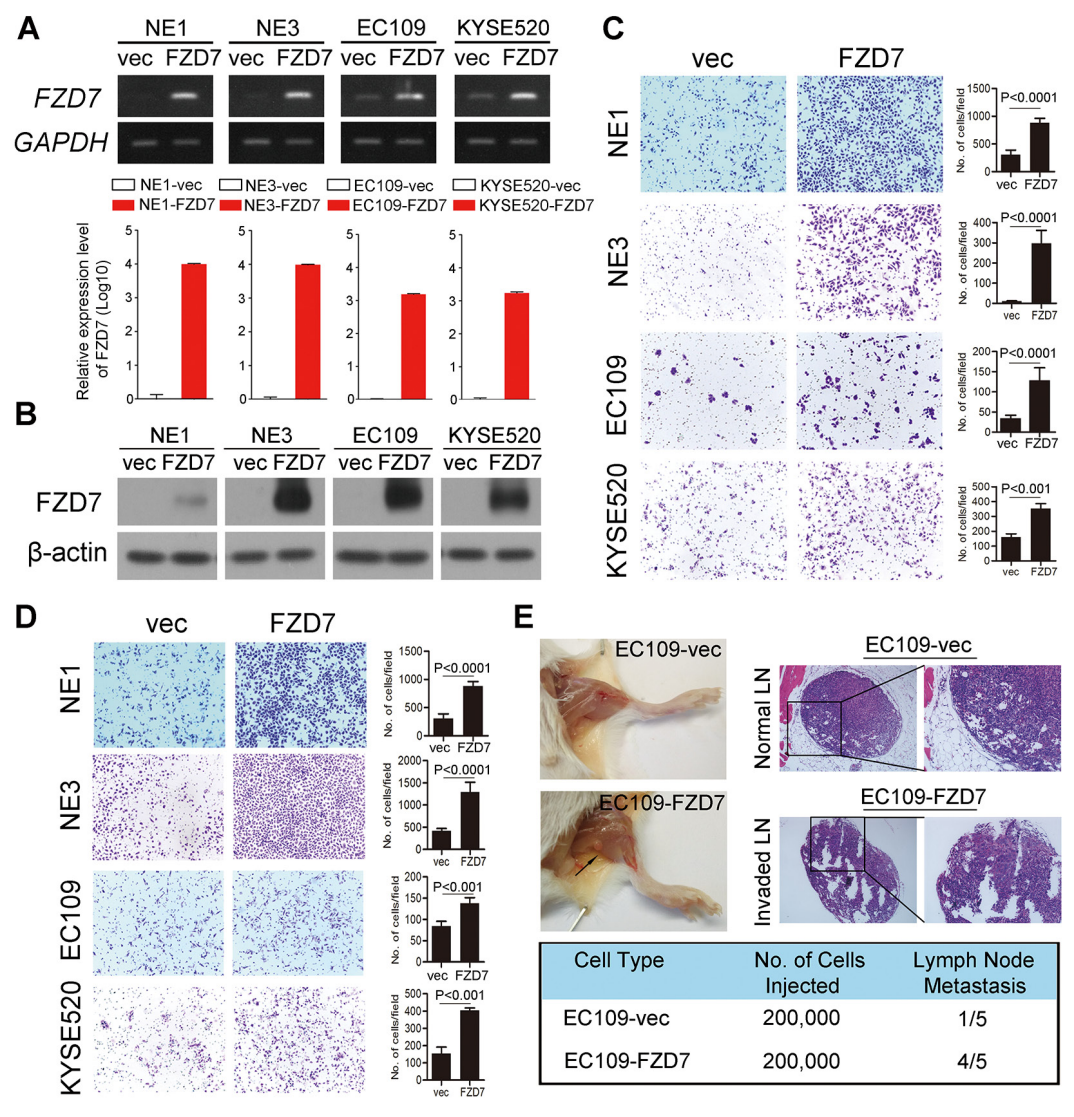
Overexpression of FZD7 promoted tumor metastasis *in vitro* and *in vivo* Expression level of FZD7 in FZD7-overexpressed cell lines was confirmed by RT-PCR and qPCR **(A)** and Western blot **(B)**. Migration assay **(C)** and invasion assay **(D)** in FZD7 overexpressed cells were represented as compared with their control cells. The average number of cells per field were shown as mean ± SD of three independent experiments. *P*<0.05 by student *t* test. **(E)** Representative images of popliteal lymph nodes (LNs) in SCID mice following subcutaneous footpad injection of the indicated EC109 clones (*Upper left*). Black arrow indicated the swollen popliteal LNs. Representative H.E. staining of normal LNs and tumor cell invaded LNs from tested animals (*Upper right*, original magnification 100x). Number of metastatic popliteal LNs in each group of animals were summarized (*Bottom*; *P* < 0.05, χ2 test).

To verify the role of FZD7 in ESCC cells, we established NE1, NE3, EC109 and KYSE520 cells which show little endogenous FZD7 transcription with FZD7 stable expression (NE1/ NE3/EC109/KYSE520-FZD7) by lentiviral transduction. Empty vector-transfected (NE1/NE3/EC109 /KYSE520-vec) cells were used as controls, respectively. The transduction efficiency was confirmed using both conventional RT-PCR and qPCR (Figure 3A), as well as Western blot analysis (Figure 3B). The results showed that the migration (Figure 3C) and invasion (Figure 3D) ability of esophageal cells with FZD7 overexpression is significantly increased compared with control cells.

In the animal experimental model, swelling popliteal lymph nodes (LNs) were observed in 4 out of 5 mice two months after the injection of EC109-FZD7 cells at the foot pads of SCID mice, whereas only one swelling popliteal LN was observed in 5 mice injected with EC109-vec cells. (Figure 3E)

### Knockdown of FZD7 could suppress ESCC cell mobility and metastasis

**Figure 4:**
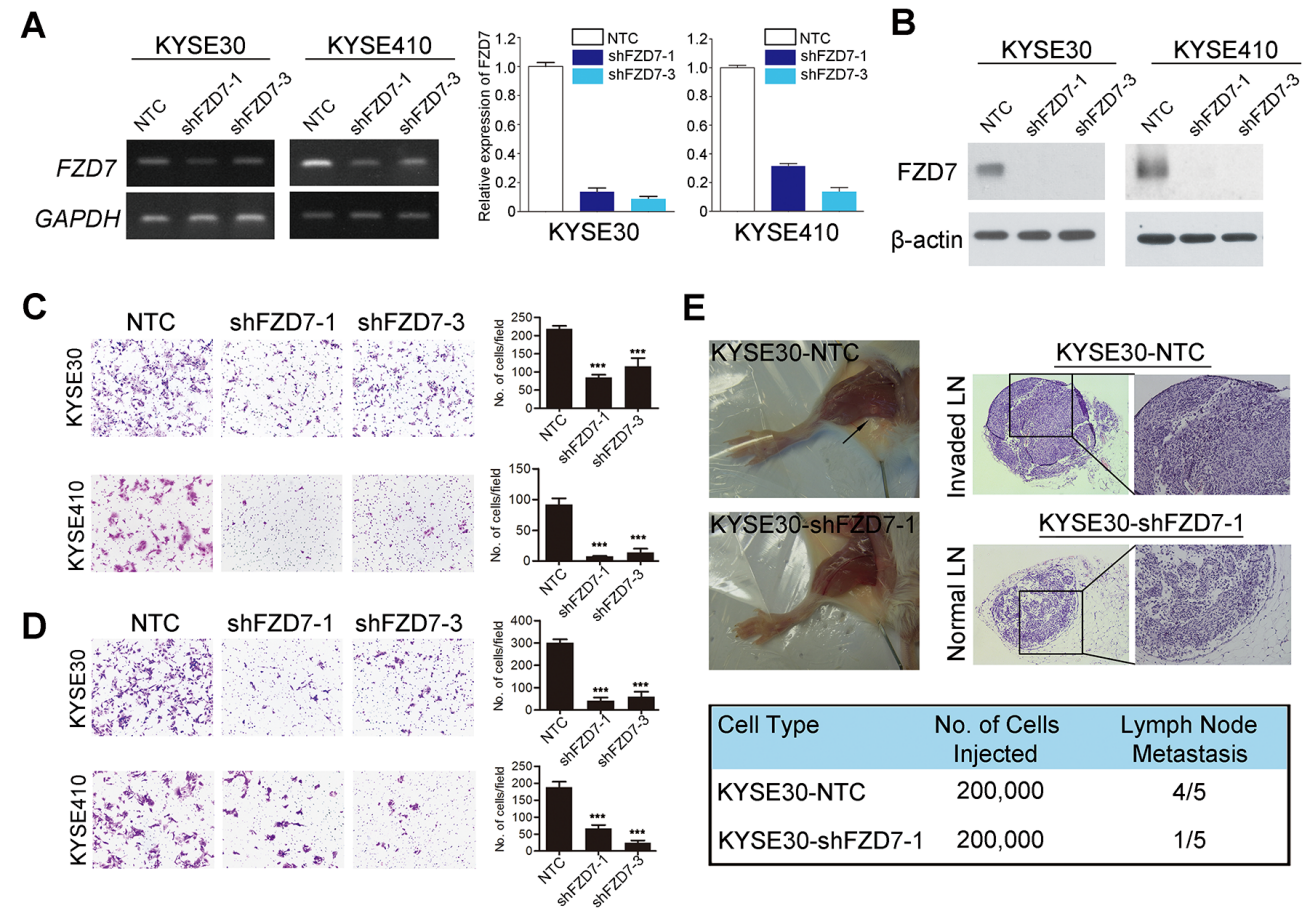
Repression of FZD7 inhibited tumor metastasis *in vitro* and *in vivo* Expression level of FZD7 in FZD7-repressed cell lines were confirmed by RT-PCR and qPCR **(A)** and Western blot **(B)**. Migration assay **(C)** and invasion assay **(D)** in FZD7 repressed cells were represented as compared with their control cells. The average number of cells per field were shown as mean ± SD of three independent experiments. *P*<0.05 by student *t* test. **(E)** Representative images of popliteal LNs in SCID mice following subcutaneous footpad injection of the indicated FZD7-repressed KYSE30 clones (*Upper left*). Representative H.E. staining of normal LNs and tumor cell invaded LNs from tested animals (*Upper right*, original magnification 100x). Number of metastatic popliteal LNs in each group of animals were summarized (*Bottom*; *P* < 0.05, χ2 test).

To further examine whether inhibition of FZD7 expression could suppress the ESCC cell mobility and tumor metastasis in mouse model, we knocked down the endogenous FZD7 expression in KYSE30 and KYSE410 cells (KYSE30/KYSE410-sh1/3) by lentiviral transduction. Non-template short hairpin RNA-transfected (KYSE30/KYSE410-NTC) cells were used as controls, respectively. Both shFZD7-1 and shFZD7-3 could efficiently decrease the mRNA (Figure 4A) and protein (Figure 4B) level of FZD7 in KYSE30 and KYSE410 cells. Cell migration and invasion assays showed that the migration (Figure 4C) and invasion (Figure 4D) ability of ESCC cells with FZD7 knockdown was obviously suppressed compared with control cells.

*In vivo* metastasis studies further showed that only one SCID mouse injected with KYSE30-shFZD7 cells showed popliteal LN metastasis, whereas LN metastasis appeared in 4 out of 5 mice injected with KYSE30-NTC cells (Figure 4E).

### Overexpression of FZD7 could induce the activity of β-catenin

**Figure 5:**
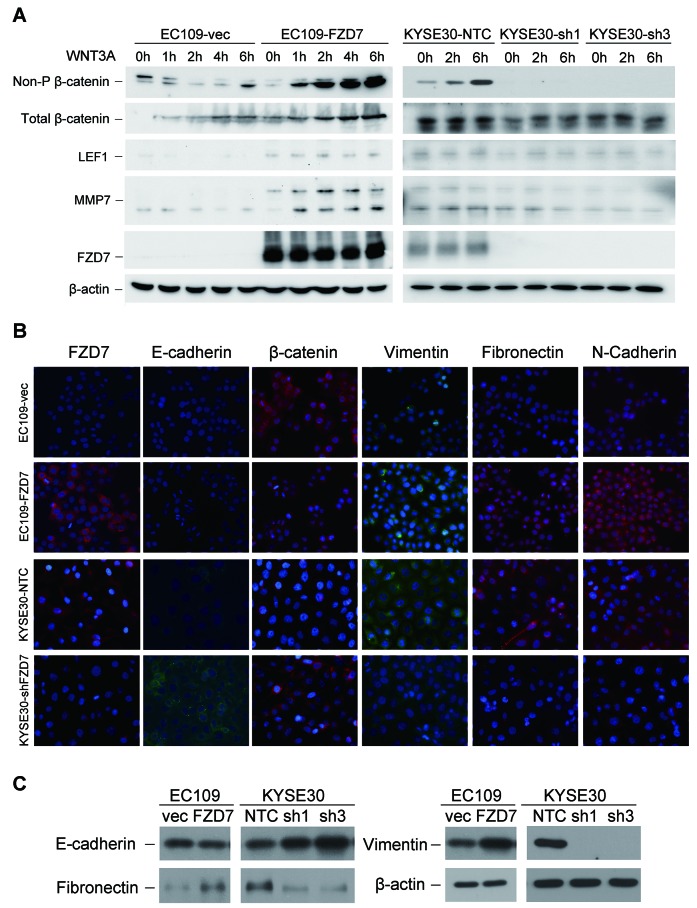
FZD7 enhances the activity of canonical WNT/β-catenin signaling with the presence of WNT3A protein and induces EMT **(A)** Activated non-phosphorylated β-catenin, total β-catenin, and two downstream targets of β-catenin LEF1 and MMP7 were detected by Western blot analysis after treatment of WNT3A protein at the indicated time-points. β-actin was used as a loading control. **(B)** Representative images showed the expression of epithelial markers and mesenchymal markers in both FZD7-oversepressing EC109 cells and FZD7-repressing KYSE30 cells (original magnification: 400x). **(C)** Epithelial markers and mesenchymal markers in both FZD7-oversepressing EC109 cells and FZD7-repressing KYSE30 cells were compared by Western Blot analysis. β-actin was used as loading control.

Previous studies have reported that FZD7 could bind with WNT3A and induce the activation of WNT signaling pathway [14]. Our data showed that, after WNT3A protein treatment, both total β-catenin and activated non-phosphorylated β-catenin in EC109-FZD7 cells were progressively increased in a time-dependent manner compared with control cells (Figure 5A). Moreover, two key downstream targets of β-catenin, LEF1 and MMP7, were also constantly increased in EC109-FZD7 cells compared with control cells. These data indicated that FZD7 may act as the receptor of WNT3A protein to induce the activation of canonical WNT/β-catenin pathway, and participate in the regulation of ESCC cell mobility and metastasis. Moreover, influence of FZD7 on the activation of β-catenin was also detected in FZD7-reppressing clones (KYSE30-FZD7sh1/sh3) and control cells (KYSE30-NTC) under WNT3A stimulation. The results showed that both non-phosphorylated β-catenin and its downstream targets such as LEF and MMP7 were repressed by knockdown of FZD7, even under WNT3A stimulation (Figure 5A). Furthermore, we detected non-phosphorylated β-catenin in both cytoplasmic and nuclear lysates of EC109-vec and EC109-FZD7 cells after WNT3A treatment. The results showed that β-catenin could accumulate into nucleus much more efficiently in EC109-FZD7 cells than control cells under WNT3A stimulation, suggesting that FZD7 could induce the canonical WNT/β-catenin pathway in ESCC cells (Supplementary Figure 3).

### FZD7 could induce epithelial-mesenchymal transition in ESCC

To determine whether the effect of FZD7 on cell migration and invasion was associated with EMT, expressions of several epithelial markers (β-catenin and E-cadherin) and mesenchymal markers (Vimentin, Fibronectin and N-cadherin) were compared between FZD7- overexpression/repression ESCC cells and their control cells by IF analysis and Western blot. In FZD7-overexpressed EC109 cells, expression of total β-catenin decreased, while all the mesenchymal markers tested were elevated. Notably, E-cadherin expression was absent in control EC109 cells so that decreased expression of E-cadherin couldn’t be detected in FZD7-overexpressed EC109 cells. On the other hand, all the epithelial markers were increased while the mesenchymal markers were decreased in FZD7-repressed KYSE30 cells (Figure 5B). The IF data were further confirmed by Western blotting (Figure 5C), strongly supporting that FZD7 could induce EMT, probably through WNT/β-catenin signaling pathway in ESCC.

## DISCUSSION

Despite the advances in diagnosis and treatment of ESCC, the 5-year survival rate after curative surgery is only 20-30%, mainly resulted from tumor metastasis, tumor recurrence and chemo-resistance [15]. How to improve the survival rate of ESCC patients and improve the life quality of them is still a difficult problem. Mounting evidences showed that the activation of WNT/β-catenin signaling pathway closely relates with cell proliferation, migration, invasion, metastasis, and poor survival of ESCC. The biological signals from WNT proteins need to be transduced into cells through receptors on the cell membrane, such as the frizzled family. However, the functions and clinical significance of FZDs in ESCC are poorly investigated.

In the present study, we found that, in 10 frizzled family members, *FZD7* was the most frequently up-regulated frizzled member in ESCC cell lines. In contrast, in the immortalized human esophageal epithelial cells NE1 and NE3, the expression level of FZD7 was quite low. Although only one study reported that FZD7 was expressed throughout normal gastrointestinal tract, from esophagus to rectum [16], the expression level of human FZD7 protein in primary ESCCs has not been addressed yet. Our results showed that protein expression level of FZD7 was frequently up-regulated in 8/10 (80%) of ESCC cell lines and 165/252 (65.5%) of ESCC patients. Importantly, up-regulation of FZD7 in ESCC clinical samples was significantly correlated with the shorter survival rate, especially that of the early stage of ESCC, and was found to be an independent prognostic marker by multivariate Cox-regression analysis. Overexpression of FZD7 has also been reported to be significantly associated with tumor cell proliferation in colorectal cancer [17] and glioma [18]. However, overexpression of FZD7 was not correlated with any clinicopathological parameters (i.e. age, sex, differentiation, LN status, TNM stage) in ESCC, which was consistent with previous findings in rental cell carcinoma [19].

Our study also showed that FZD7 could significantly promote cell migration/ invasion *in vitro* and metastasis *in vivo*. ESCC cells with enforced FZD7 expression showed obvious higher ability of migration and invasion. In contrast, shRNA-mediated silencing of FZD7 expression could suppress its metastasis-promoting ability. However, either overexpression or knockdown of FZD7 did not affect cell growth rate of ESCC cell lines (data not shown). In the animal model for tumor metastasis, introduction of exogenous FZD7 in ESCC cells could result in increased popliteal lymph nodes metastasis. Previous studies showed that FZD7 might drive aggressiveness in Stem-A Ovarian cancer by regulating cell proliferation, cell cycle progression, maintenance of the Mesenchymal phenotype and cell migration via casein kinase 1-mediated non-canonical WNT/PCP pathway [20]. In hepatocellular carcinoma, upregulation of FZD7 was positively correlated with nuclear accumulation of wild-type β-catenin [21, 22], and interaction between WNT3 and FZD7 could lead to activation of canonical WNT signaling pathway [14]. Down-regulation of FZD7 in cervical cancer cells could inhibit cell invasion and migration by inhibiting the expression level and activities of MMP2 and MMP9, as well as inhibiting the expression of phosphorylated JNK and c-jun [23]. In addition, microRNAs such as microRNA-542-3p, microRNA-184, and microRNA-199a-5p could target FZD7 and suppress cell proliferation [24–26].

To further look for the mechanism underlying the metastasis-promoting function of FZD7 in ESCC, we cultured ESCC cell lines with WNT3A conditioned medium. WNT3A has been reported as a typical ligand of FZD7. Our results showed that FZD7 could effectively increase the expression level of active form of β-catenin. In addition, two of its key downstream targets (LEF1 and MMP7) were also activated, suggesting that FZD7 could transmit the canonical WNT signals through β-catenin to the downstream molecules. This result was in line with previous study that β-catenin could translocate into the nucleus and interact with the LEF1 protein to activate downstream targets via the canonical WNT-FZD7 signaling [27]. MMP7 is also an important downstream target of the canonical WNT/β-catenin signaling pathway. We also confirmed that the active form of β-catenin was obviously accumulated into the nucleus of FZD7-overexpressed cells under WNT3A treatment. These data indicated that overexpression of FZD7 indeed activates the WNT/β-catenin signaling pathway and results in the biological alterations relevant with tumor metastasis.

Notably, although FZD7 was reported to promote cell proliferation in a series of human solid cancers, it was not a growth-promoting factor in ESCC. One of the possible reasons was that function of the *FZD7* gene may vary in different types of tissues or organs. Furthermore, the role of FZD7 in ESCC metastasis has been clearly addressed. However, how FZD7 affects the expression of LEF1 and MMP7 needs to be further addressed. Our study also found that the EMT process was activated by forced expression of FZD7, whereas the EMT process was inhibited by depletion of FZD7 expression in ESCC cells. WNT signaling could promote EMT by inhibiting glycogen synthase kinase-3β (GSK3β) to stabilize β-catenin, which translocates to the nucleus to engage the transcription factors lymphoid enhancer-binding factor 1 (LEF1) and T cell factor (TCF) and promote a gene expression program that favours EMT [28].

In conclusion, our study provided first evidence that FZD7 is an important factor in ESCC metastasis, and could induce EMT and WNT/β-catenin signaling pathway. FZD7 may serve as a novel prognostic marker and a potential therapeutic target for esophageal cancer patients.

## MATERIALS AND METHODS

### ESCC clinical samples

All of the ESCC specimens (tumor and adjacent non-tumor tissues) were collected immediately after surgical resection at Cancer Center of Sun Yat-sen University (Guangzhou, China). None of these patients received preoperative treatment. These tissues with clinical information were used to produce tissue microarray (TMA) blocks, which were constructed according to standard procedure. Multiple sections (5um thick) were used to perform immunohistochemical (IHC) analysis. Tumor stages of the specimens on the tissue microarray were categorized according to the tumor-node-metastasis (TNM) system by American Joint Committee on cancer (AJCC) [29].

### Cell lines

Immortalized human esophageal epithelial cells NE1 and NE3 were established in Professor George Tsao’s laboratory (Department of Anatomy, The University of Hong Kong). Chinese ESCC cell lines EC109, HKESC1, EC9706 and EC18 were kindly provided by Professor Srivastava (Department of Pathology, The university of Hong Kong). Six Japanese ESCC cell lines KYSE30, KYSE140, KYSE180, KYSE410, KYSE510 and KYSE520 were obtained from DAMZ, the German Resource Center for Biological Material [30].

### Establishment of stable cell lines

The full length of FZD7-ORF was amplified and cloned into lentiviral expression vector pLenti6 (Sigma-Aldrich, St. Louis, Missouri). Plasmids reconstructed with shRNAs targeting FZD7 were obtained from Sigma-Aldrich (St. Louis, Missouri). The sequences of these shRNAs have been listed in Supplementary Table 2. Lentivirus were produced using transfection reagent ScreenFect A (Incella, Germany) and were applied to infect ESCC cells according to the manufacturer’s instructions. The stable cell lines were screened with puromycin in proper concentrations.

### RNA extraction and quantitative real-time PCR

Total RNA was extracted using TRIZOL Reagent (Invitrogen, Carlsbad, CA) and reverse transcription polymerase chain reaction (RT-PCR) was performed using PrimeScript^TM^ RT Master Mix (Takara, Japan) according to the manufacturer’s instructions. For quantitative real-time PCR (qPCR), the cDNA was amplified using SYBR Green (Applied Biosystems, Carlsbad, CA) and an ABI PRISM 7900 Sequence Detector. Data was analyzed using SDS v2.4 software (Applied Biosystems). All of the qPCR reactions were performed in triplicates. Housekeeping gene *GAPDH* was used as an internal control. All of the primers used in this study were synthesized by Tech Dragon (Hong Kong) (Supplementary Table 3)

### Immunohistochemistry (IHC) staining

The ESCC TMA slides were used to perform IHC staining following the manufacture’s instruction (Dako, Denmark) to detect the expression level of FZD7 protein. The antibody was purchased from Abcam (Cambridge, UK). The results of IHC staining were viewed and scored separately by two independent investigators without any prior knowledge of clinicopathologic data. Expression of FZD7 was scored as absent (total absence of staining), very weak (faint staining), moderate staining, or strong staining, and multiply by the percentage of positively-stained tumor cells. The scores of FZD7 expression in each sample were between 0 and 300. After calculation, we used ROC curve in SPSS to get a cut-off value of 192.5. And the samples which got a score higher than the cut-off value were defined as “high expression”, while the samples with a score lower than the cut-off value were defined as “low expression”. The results of IHC staining were imaged using SPOT imaging software (Nikon) under microscope.

### Western blot analysis

Western blot analyses were performed following the standard protocol. Human-anti-FZD7 antibody was kindly provided by Dr. Christina Wu from the University of California San Diego. Other antibodies used in this paper were obtained from Cell Signaling Technology (Danvers, MA), Abcam and Proteintech (Rosemont, IL) (Supplementary Table 4).

### Cell migration and invasion assay

Cell ability of migration and invasion were performed using 24-well migration chamber (Corning, NY) or 24-well BioCoat Matrigel Invasion Chambers (BD Biosciences, CA) with polyethylene terephthalate (PET)-based membrane of 8.0-um pore size. In Brief, 1x10^5^ cells were suspended with serum-free culture medium and seeded in the top chambers. Complete culture medium with 10% FBS was used in the bottom chambers. The chambers were collected after 24/48 hours’ incubation. Cells which moved through the membrane were fixed with 4% PFA and stained with 0.5% crystal violet solution. The number of cells invaded through the membrane per field were counted and imaged using SPOT imaging software (Nikon) under microscope.

### *In vivo* lymph node metastasis assay

The study protocol was approved by and performed in accordance with the Committee of the Use of Live Animals in Teaching and Research at Shenzhen University School of Medicine. EC109-FZD7/vec and KYSE30-NTC/shFZD7 cells (2x10^5^) were injected subcutaneously into foot pads of 4-week-old male SCID mice (n=5), respectively. Mice were examined for tumor growth in the foot pads every 3 days for 2 months. Following sacrifice, popliteal lymph nodes were collected, fixed in 4% PFA and embedded in paraffin block for H&E assay.

### Immunofluorescence (IF) staining

Cells were grown on glass coverslips up to 80% confluence. Then the cell layers were washed with 1X PBS and fixed with 4% PFA and processed for IF staining. The cells were firstly incubated with primary antibodies overnight at 4 °C. After thorough washing, cells were incubated with fluorescence-conjugated secondary antibodies for 1h at room temperature. Finally, cells were washed and stained with DAPI (Thermo Fisher Scientific, MA) for 10 min at room temperature avoid light. The slides were covered with mounting medium and images were captured under Leica DMRA fluorescence microscope (Wetzlar, Germany). Antibodies used for IF staining have been listed in Supplementary Table 4.

### Nucleocytoplasmic separation experiment

About 2x10^6^ cells were seeded one day in prior in about 80% confluence. Then the cells were cultured with fresh complete culture medium or WNT3A conditioned medium for 6h. After that, the Nucleocytoplasmic separation experiment was performed using Nucleocytoplasmic Separation Kit following the manufacture’s instruction (Beyotime, China). Briefly, the cells were washed with cold normal saline for twice, then were scraped off and collected to perform the nucleocytoplasmic separation procedure. The nuclear lysates and cytoplasmic lysates were denaturalized and applied to perform western blot analysis following the standard protocol.

### Statistical analysis

All of statistical analyses were carried out with APSS for Windows, version 16.0 (SPSS Inc., Chicago, IL). The correlation between FZD7 expression and clinical-pathological characteristics of ESCC patients was analyzed by the Pearson χ ^2^ test. Survival curves were generated according to the Kaplan-Meier method, and statistical analysis was performed using the log-rank test. Univariable and multivariable Cox proportional hazard regression models were used to analyze independent prognostic factors. Paired Student’s t-test and ANOVA with post hoc test were used for most studies in the figure legends. All of the experiments were repeated for at least three times, and the data were expressed as means ± SD. The P values were denoted as * *P* <0.05, ** *P*< 0.001, and *** *P*< 0.0001 in all figures.

## SUPPLEMENTARY MATERIALS FIGURES AND TABLES


