# Supplementary material for: FZD7 is a novel prognostic marker and promotes tumor metastasis via WNT and EMT signaling pathways in esophageal squamous cell carcinoma
Source: Oncotarget. 2017 Jul 26;8(39):65957–68. doi: 10.18632/oncotarget.19586 (PMC5630385; doi:10.18632/oncotarget.19586)
Supplement: Supplementary file 1 [file oncotarget-08-65957-s001.pdf]

FZD7 is a novel prognostic marker and promotes tumor metastasis via WNT and EMT signaling pathways in esophageal squamous cell carcinoma

SUPPLEMENTARY MATERIALS

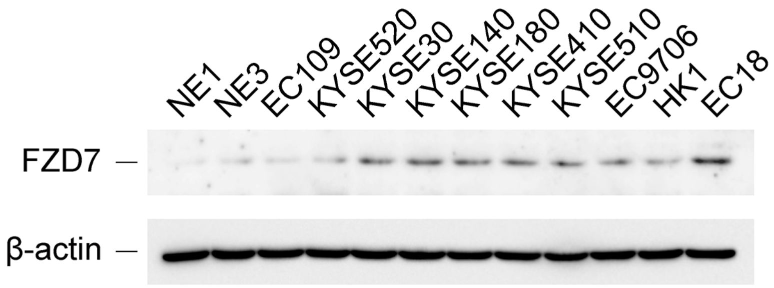

Supplementary Figure 1: Protein expression level of FZD7 in immortalized normal esophageal epithelial cell lines and ESCC cell lines. **β-actin was used as loading control.**

A

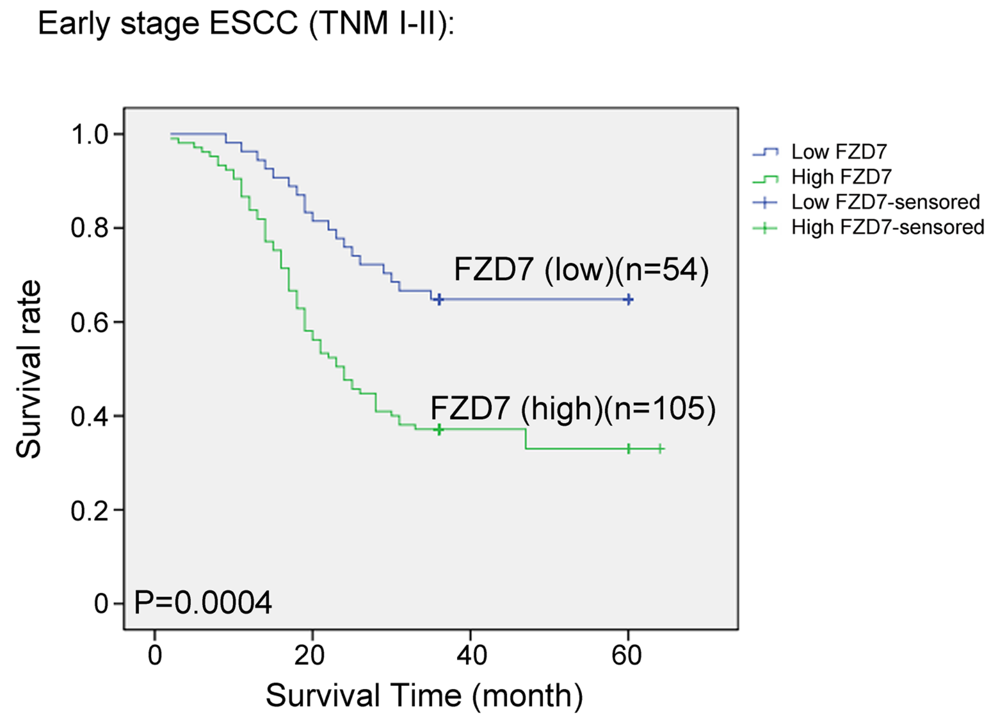

B

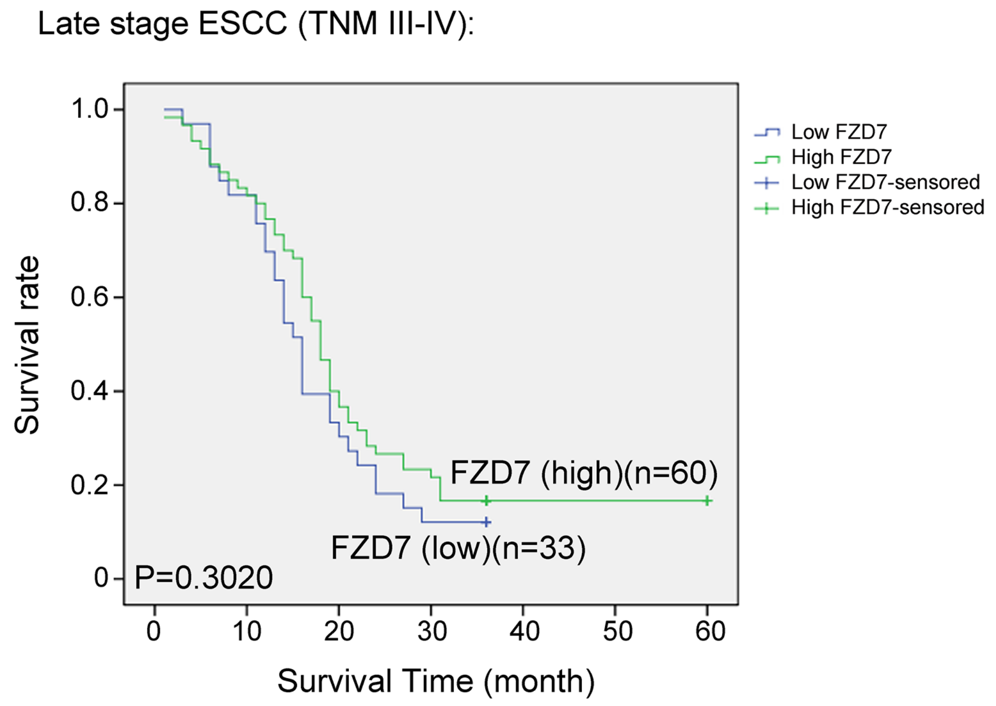

Supplementary Figure 2: Kaplan-Meier Survival analysis according to FZD7 expression level in early stage and late stage of ESCC patients.

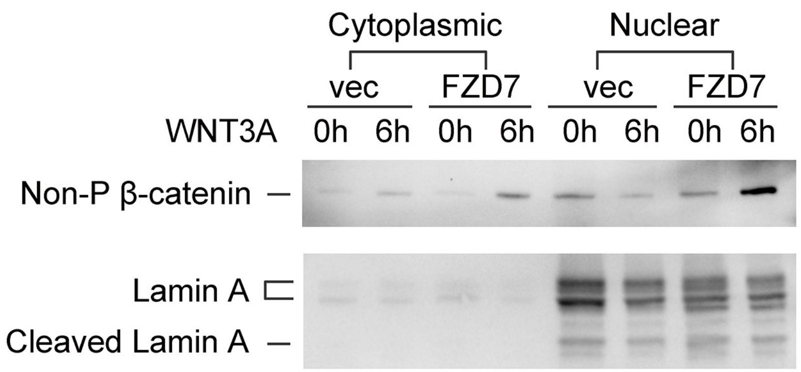

Supplementary Figure 3: Expression level of non-phosphorylated β-catenin (Non-P β-catenin) in cytoplasm and nucleus of EC109-vec/FZD7 cells under WNT3A protein stimulation. Lamin A was used as loading control.

Supplementary Table 1: Correlation between FZD7 expression and clinico-pathological features in primary ESCCs

Clinico-pathological features	Number	FZD7 overexpression	*P* value
**Gender**			
Female	112	74 (66.1%)	
Male	140	91 (65.0%)	0.859
**Age**			
<60	132	82 (62.1%)	
≥60	120	83 (69.2%)	0.240
**Tumor size (cm)**			
<30	204	131 (64.2%)	
≥30	48	34 (70.8%)	0.386
**Lymph node metastasis**			
Absent	134	87 (64.9%)	
Present	118	78 (66.1%)	0.845
**Differentiation**			
Well	64	41 (64.1%)	
Medium	124	85 (68.5%)	
Poor	64	39 (60.9%)	0.561
**Depth of invasion**			
0-2	89	59 (66.3%)	
3-4	163	106 (65.0%)	0.840
**Tumor stage (TNM)**			
Stage I~II	159	105 (66.0%)	
Stage III~IV	93	60 (64.5%)	0.806

Supplementary Table 2: shRNA sequences targeting FZD7

ShRNA name	Targeted region of *FZD7* gene	ShRNA Sequence (5’→3’)
ShFZD7-1 (TRCN0000357011)	CDS	CCGGCAACGGCCTGATGTACTTTAACTCGAGTTAAAGTACATC AGGCCGTTGTTTTTG
ShFZD7-3 (TRCN0000367752)	3’ UTR	CCGGGGGCCTGTTTCTGTAACTTTCCTCGAGGAAAGTTACAGA AACAGGCCCTTTTTG

Supplementary Table 3: Primer list

Primer name	Primer sequence (5’→ 3’)
FZD1-QF	GGGGCTTAACAACGTGGAC
FZD1-QR	CAGAAAGGACGTGCCGATAAA
FZD2-QF	GTGCCATCCTATCTCAGCTACA
FZD2-QR	CTGCATGTCTACCAAGTACGTG
FZD3-QF	AATATGGACGTGTCACACTTCC
FZD3-QR	GGATATGGCTCATCACAATCTGG
FZD4-QF	CCTCGGCTACAACGTGACC
FZD4-QR	TGCACATTGGCACATAAACAGA
FZD5-QF	CCGTTCGTGTGCAAGTGTC
FZD5-QR	GAAGCGTTCCATGTCGATGAG
FZD6-QF	GCGATAGCACAGCCTGCAATA
FZD6-QR	AATGGTAAGAATCACCCACCAC
FZD7-QF	CAGACGTGCAAGAGCTATGC
FZD7-QR	ACGATCATGGTCATCAGGTACT
FZD8-QF	TACAACCGCGTCAAGACAGG
FZD8-QR	CCATGTCGATAAGGAAGGTGGAG
FZD9-QF	TGCGAGAACCCCGAGAAGT
FZD9-QR	GGGACCAGAACACCTCGAC
FZD10-QF	GCTCATGGTGCGTATCGGG
FZD10-QR	GAGGCGTTCGTAAAAGTAGCA
GAPDH-QF	CATGAGAAGTATGACAACAGCCT
GAPDH-QR	AGTCCTTCCACGATACCAAAGT

Supplementary Table 4: Antibody list

The antibodies used in this study were as below

Antibody Name	Company	Catolog No.	Application
FZD7	Abcam	ab64636	IF (1/100 dilution)<br>IHC (1/50 dilution)
Non-phospho<br>β-Catenin	Cell signaling Technology	19807	IF (1/100 dilution)<br>WB (1/1000 dilution)
Total β-Catenin	Cell signaling Technology	9587	WB (1/1000 dilution)
E-Cadherin	Abcam	Ab76055	IF (1/100 dilution)
E-Cadherin	Cell signaling Technology	3195	WB (1/1000 dilution)
Vimentin	Cell signaling Technology	5741	IF (1/100 dilution)<br>WB (1/1000 dilution)
Fibronectin	Proteintech	15613-1-AP	IF (1/100 dilution)<br>WB (1/1000 dilution)
N-Cadherin	Cell signaling Technology	13116	IF (1/100 dilution)
LEF1	Cell signaling Technology	2230	WB (1/1000 dilution)
MMP7	Cell signaling Technology	3801	WB (1/1000 dilution)
Lamin A/C	Cell signaling Technology	2032	WB (1/1500 dilution)
